# Dysregulation of Protein Kinase C in Adult Depression and Suicide: Evidence From Postmortem Brain Studies

**DOI:** 10.1093/ijnp/pyab003

**Published:** 2021-01-30

**Authors:** Ghanshyam N Pandey, Anuradha Sharma, Hooriyah S Rizavi, Xinguo Ren

**Affiliations:** University of Illinois at Chicago, Department of Psychiatry, Chicago, IL, USA

**Keywords:** PKC, PKC isozymes, depression, suicide, postmortem brain, prefrontal cortex

## Abstract

**Background:**

Several lines of evidence suggest the abnormalities of protein kinase C (PKC) signaling system in mood disorders and suicide based primarily on the studies of PKC and its isozymes in the platelets and postmortem brain of depressed and suicidal subjects. In this study, we examined the role of PKC isozymes in depression and suicide.

**Methods:**

We determined the protein and mRNA expression of various PKC isozymes in the prefrontal cortical region (Brodmann area 9) in 24 normal control subjects, 24 depressed suicide (DS) subjects, and 12 depressed nonsuicide (DNS) subjects. The levels of mRNA in the prefrontal cortex were determined by quantitative real-time reverse transcription PCR, and the protein expression was determined by western blotting.

**Results:**

We observed a significant decrease in mRNA expression of PKCα, PKCβI, PKCδ, and PKCε and decreased protein expression in either the membrane or the cytosol fraction of PKC isozymes PKCα, PKCβI, PKCβII, and PKCδ in DS and DNS subjects compared with normal control subjects.

**Conclusions:**

The current study provides detailed evidence of specific dysregulation of certain PKC isozymes in the postmortem brain of DS and DNS subjects and further supports earlier evidence for the role of PKC in the platelets and brain of the adult and teenage depressed and suicidal population. This comprehensive study may lead to further knowledge of the involvement of PKC in the pathophysiology of depression and suicide.

**Significance Statement**

Abnormalities of serotonin and norepinephrine as well as their receptors such as 5-HT, α-adrenergic, and β-adrenergic receptors have been implicated in the pathophysiology of depression and suicide. However, their biochemical and behavioral consequences are not very clear. These receptors produce their biochemical effects by downstream signaling through signaling pathways to which they are linked, such as phosphoinositide (PI) and adenylyl cyclase signaling. PKC is an important component of PI signaling to which 5-HT_2A_ receptors are linked. Our study of postmortem brain (prefrontal cortex, PFC) indicated a decrease in some PKC isozymes. These specific isozymes perform different biological functions in the brain. Our studies of PKC isozymes are significant because they show abnormalities of specific isozymes in depression and suicide and may provide specific therapeutic targets for the development of newer therapeutics and more appropriate treatments of those patients who do not respond to standard antidepressant treatment.

## Introduction

Suicide is a major public health concern as 44 000 people die of suicide each year in the United States alone (O’Rourke et al., 2020). It is the second -most prevalent cause of death in young adults and fourth in adults 35–54 years old (NIMH, 2020). A consistent rise in suicide rates has been seen in the United States over the last 10–15 years (APA, 2020). Depression is a major risk factor for suicide and suicidal behavior (Ng et al., 2017), and in terms of prevalence, depression itself is estimated to by the World Health Organization be the second largest contributor (with a prevalence rate of 10.8% during the lifetime of the general population) to global burden of disease (Lim et al., 2018).

There are many psychological and demographic studies of suicidal behavior; however, neurobiological studies of suicide are few. Earlier studies of neurobiological mechanisms associated with depression and suicide were mainly focused on neurotransmitter levels and neurotransmitter receptors, resulting in the amine hypothesis of depressive illness (Bunney and Davis, 1965). Subsequently, the role of neurotransmitter receptor and receptor signaling was studied, such as the protein kinase A – adenylyl cyclase signaling system, mitogen-activated protein kinase signaling system, and glycogen synthase kinase-3β and phospholipase C (PLC) – phosphoinositide (PI) signaling system (Shelton, 2007; Carballo et al., 2008; Pandey, 2011). The main neurobiological mechanism (i.e., amine hypothesis associated with suicidal behavior) was primarily focused on the abnormalities of serotonin (5-hydroxytryptamine [5-HT]) and serotonin receptors, known as the serotonin hypothesis (Mann, 1999; Carballo et al., 2008; Albert and Benkelfat, 2013). For example, it was shown by some investigators that the levels of 5-hydroxyindoleacetic acid, a metabolite of serotonin, was significantly reduced in the cerebrospinal fluid of suicidal compared with nonsuicidal patients and was suggested as a suicide risk predictor (Cooper et al., 1992; Nordström et al., 1994). Subsequently, it was also shown that a subtype of serotonin receptor, known as 5-HT_2A_ receptor, was significantly higher in the platelets of suicidal patients compared with nonsuicidal patients (Pandey et al., 1995). It was also reported that 5-HT_2A_ receptor was also significantly increased in the postmortem brain of suicidal subjects (Stanley and Mann, 1983; Pandey et al., 2002b).

5-HT_2A_ receptors are linked to the PLC-PI signaling system. Activation of this signaling cascade by agonists of 5-HT_2A_ receptor such as serotonin along with other receptors such as α-adrenergic, M1 muscarinic, and 5-HT_2C_ receptors have been shown by some studies to be abnormal in depression and suicide (Akin et al., 2004; Pandey et al., 2006). For example, the levels of PLCβ _1_ and PLC activity were shown to be reduced in teenage suicide subjects (Pandey et al., 1999). These studies suggest the PI-PLC signaling system as an important signaling pathway, abnormalities of which have been implicated in the pathophysiology of depression and suicide. In the last decade, different research groups studying the neurobiology of suicide and depression have been interested in different components of this pathway, particularly protein kinase C (PKC).

Some of our earlier studies were focused on different components of the PI signaling system. Besides PLC, PKC is another important component of this system, as described previously (Pandey et al., 2002a, 2004). The activation of PI-PLC signaling results in the formation of inositol 1,4,5 triphosphate (IP_3_), which facilitates the release of calcium on stimulation, whereas in the other arm of this signaling system, the formation of diacylglycerol (DAG) activates PKC (Cocco et al., 2015). Activation of PKC involves the conversion of the latent auto-inhibitory form of PKC in the cytosol to the active PKC and its transition to the membrane (Igumenova, 2015). The role of calcium in the pathophysiology of bipolar (BP) disorders and depression has been studied, and some of the calcium-related genes, such as CACNA1C, have been reported to be associated with the development of BP illness and depression (Green et al., 2010). All of these studies therefore point to the direct or indirect role of PKC in suicide and depression.

This study therefore aimed to investigate alterations in PKC isozymes in depressed suicide (DS) and depressed nonsuicide (DNS) subjects. We determined the mRNA and protein expression of PKC isozymes as well as the effect of other confounding factors in this set of experimental subjects. In our previous studies, we found altered PKC expression and altered PKC activity and downstream signaling in teenage suicide subjects (Pandey et al., 1997). In this study, we were interested to examine whether the current findings in adult DS and DNS subjects are similar to the results obtained earlier in teenage suicide subjects.

## Materials and Methods

### Subjects, Diagnostic Procedure, and Criteria for Inclusion/Exclusion

The current study was performed on prefrontal cortex (PFC) region (Brodmann area 9 [BA9]) of DS (n = 24), DNS (n = 12), and nonpsychiatric normal control (NC, n = 24) subjects. The postmortem brains were obtained from the Maryland Brain Collection at the Maryland Psychiatric Research Center, and samples were collected after informed written consent and detailed interview of at least 1 family member of the deceased. The interviews were conducted by a trained psychiatrist, and brain tissue from all subjects was thoroughly examined by experienced neuropathologists. Blood and urine samples were used to access the toxicology details. All procedures were approved by the University of Maryland Institutional Review Board and by the University of Illinois at Chicago Institutional Review Board.

Diagnosis was performed according to Structured Clinical Interview for DSM-IV criteria (First et al., 1997). For the same, the detailed interviews of family members, toxicology data, medical records of the case, and other records from the office of the medical examiner were analyzed by 2 psychiatrists, and their consensus report was considered as final diagnosis. The individual subjects diagnosed with major depressive disorder (MDD) who committed suicide were included in the DS group, and the subjects diagnosed with MDD who did not commit suicide were included in the DNS group. The consensus diagnostic report ensured that no NC subjects had any mental illness. The subjects with a history of substance abuse, family history of psychiatric illness, accidental deaths, or death after prolonged hospitalization were excluded from the NC group. Additionally, none of the subjects with any other mental illness, major history of medical or neurological disorders, and HIV were included in any of the study groups. All subjects were considered without any sexual or ethnic differentiation. Detailed characteristics of the subjects of all 3 groups are listed in Supplementary Table 1.

### mRNA Expression Studies

RNA extraction and quantification were performed as described by Pandey et al. (2019). Briefly, total RNA was extracted using TRIZOL (Invitrogen, Thermo Fisher Scientific, Waltham, MA; Life Technologies Corporation, Carlsbad, CA) method followed by DNase treatment. After quantitation and purity assessment using Nanodrop ND-1000 (Nano Drop Thermo Fisher Scientific, Waltham, MA) and Agilent Bioanalyzer 2100 (Agilent Technologies, Santa Clara, CA), 1 μg RNA of each sample was reverse transcribed to obtain cDNA in GeneAmp PCR system (Applied Biosystems, Foster City, CA) using random hexamers (50 ng), RNase out (10 units), and M-MLV reverse transcriptase enzyme (200 units) (Invitrogen) as per instructions by the manufacturer (described in Pandey et al., 2019). The cDNA was diluted 1:10 using diethyl pyrocarbonate (DEPC)-treated water (Ambion, Inc., Austin, TX) and stored at 4°C for further analysis. All RNA samples considered for cDNA preparation had values 1.8–2.0 for 260/280 nm and ≥5.5 for RNA integrity number (RIN).

Further diluted cDNA was used for quantitative PCR using the MX3005p sequence detection system from Agilent. Taqman primers of all PKC isozymes for gene expression assays (Applied Biosystems) are listed in Table 1. Glyceraldehyde 3-phosphate dehydrogenase (GAPDH) and β-actin (ACTB) were used as house-keeping genes [found as suitable control genes for our current data set after extensive testing as described in Pandey et al. (2019)]. To eliminate nonspecific amplification events, no template and no MMLV (reverse transcriptase) enzyme controls were included in each plate. mRNA expression of each isozyme was normalized to geometric mean of housekeeping genes and analyzed using Livak’s Method (2^−∆∆Ct^) control samples. Results are expressed as fold change (FC) compared with control.

**Table 1.** List of Taqman Probes Used for Gene Expression Assay

Genes	Taqman accession no.	Probe location (exon boundary)	Assay function
ACTB	Hs99999903_m1	1-1	House Keeping (HK)
GAPDH	Hs99999905_m1	3-3	HK
PRKCA (PKCα)	Hs00176973_m1	11–12	Target gene
PRKCB1 (PKCβI)	Hs01030676_m1	16–17	Target gene
PRKCB2 (PKCβII)	Hs01034075_m1	16–17	Target gene
PRKCG (PKCγ)	Hs00177010_m1	3–4	Target gene
PRKCD (PKCδ)	Hs00178914_m1	18–19	Target gene
PRKCE (PKCε)	Hs00178455_m1	5–6	Target gene

### Protein Expression Studies

Cytosol and membrane fractions of protein were isolated from 100 mg tissue of PFC region, and changes in protein expression were studied using the protocol as described by (Dwivedi and Pandey, 1999). Briefly, 30 μg protein of cytosol and membrane fraction of each sample was electrophorized on 7.5% (w/v) agarose gel followed by electrophoretic transfer of separated protein bands on nitrocellulose membrane (Amersham, IL). Blotted membrane was blocked with TBST (1X Tris-buffered saline, 0.1% Tween 20 detergent for Western blotting) buffer containing 5% skimmed milk followed by monoclonal anti-PKC (α, βI, βII, γ, δ or ε) primary antibody (1:3000) incubation overnight at 4°C. The next day, the blot was washed with TBST (1X Tris-buffered saline, 0.1% Tween 20 detergent for Western blotting) and incubated with HRP (horseradish peroxide) conjugated secondary antibody (1:5000). Detection of protein of interest was carried out by exposing the blot to enhanced chemiluminescent film. Each membrane was then incubated in ReBlot Plus Mild Antibody Stripping Solution (Chemicon®, Thermo Fisher Scientific, Waltham, MA) and reprobed for detection of β-actin. The autoradiogram was captured, and optical density of bands was analyzed using Loats Image Analysis System (Westminster, MD). Each protein was normalized with house-keeping gene β-actin, and results were plotted as relative protein expression (in terms of percentage change [PC]) compared with control.

### Statistical Analysis and Effect of Confounding Variables

The data obtained in the current study were analyzed using IBM SPSS Statistics Software version 25 (IBM Tech, NY). This analysis was individually conducted in the PFC region of the brain for both mRNA and protein to determine the difference of PKC isoform levels between the subjects. Means of demographical parameters—age, brain pH, and postmortem interval (PMI)–were compared using 1-way ANOVA with Bonferroni post-hoc test, whereas differences in race and gender of the 3 groups were analyzed using Pearson’s chi-square test. To study the overall effect in multiple comparisons, all 3 groups were analyzed jointly using the multivariate analysis of covariance (MANCOVA) and adjusting the effects of confounding variables age, PMI, and brain pH as covariates. For multiple comparisons, we also performed Bonferroni correction and used post-hoc *t* test for paired comparisons. *P* ≤ .05 was considered a statistically significant difference.

## Results

### Human Subjects and Associated Demographics

The demographic, toxicological, and clinical characteristics of the human adult subjects divided into the NC (n = 24), DS (n = 24), and DNS (n = 12) groups are given in (Table 2; Supplementary Table 1). There was no significant difference observed in average age (*P* = .52 and *P* = .39) and PMI (*P* = .19 and *P* = .94) for DS and DNS subjects compared with NC subjects. There was a significant difference in pH between NC and DNS subjects (*P* = .002) but not between NC and DS subjects (*P* = .87). Diagnosis (i.e., NC, DS, or DNS) was independent of the race and gender (df [2] = 2.134, *P* = .344) of the subjects.

**Table 2.** Demographic Characteristics of Subjects

Parameter	NC	DS	DNS	Significance
Subjects (n)	24	24	12	NA
Age	42.08 ± 15.35	38.95 ± 15.39	49.50 ± 17.17	.175 (*P*1 = 1.00, *P*2 = .563)
Race	B:7, W:17	B:2, W:22	B:3, W:9	.288
Gender	M:20, F:4	M:14, F:10	M:7, F:5	.239
Postmortem interval (h)	16.54 ± 6.5	18.91 ± 6.02	17.91 ± 9.31	.502 (*P*1 = .731, *P*2 = 1.00)
Brain pH	7.01 ± 0.15	6.96 ± 0.25	6.8 ± 0.269	.026 (*P*1 = 1, *P*2 = .022)
Drug or alcohol overdose	0	10	0	NA
Antidepressants	0	16	11	NA

Abbreviations: B, Black; DNS, depressed nonsuicide subjects; DS, depressed suicide subjects; F, female; M, male; NC, normal control subjects; W, White.

*P*1 and *P*2 = *P* values for multiple comparison of DS and DNS with NC, respectively, in 1-way ANOVA. Values are presented as mean ± SD.

### mRNA Expression

To examine depression- and suicide-associated alterations in the expression of PKC isozymes at the transcriptional level, we studied total mRNA levels of PKCα, PKCβI, PKCβII, PKCγ, PKCδ, and PKCε. The overall group effect using the MANCOVA model with Bonferroni post-hoc test showed that the mRNA expression of PKCα (F[5,60] = 5.031, *P* = .001), PKCβII (F[5,60] = 5.170, *P* = .001), and PKCε (F[8,58] = 3.494, *P* = .008) differed significantly between the 3 groups. However, these 3 groups did not differ significantly in the mRNA expression of PKCβI, PKCγ, and PKCδ. Other confounding variables such as age, race, PMI, and gender showed no significant effects on any of the study groups. We further performed pairwise comparisons using post-hoc *t* test (*P* ≤ .05) for DS and DNS groups compared with the NC group and Bonferroni correction (*P* ≤ .008) to fix multiple group comparison type I errors.

**Figure 1.**
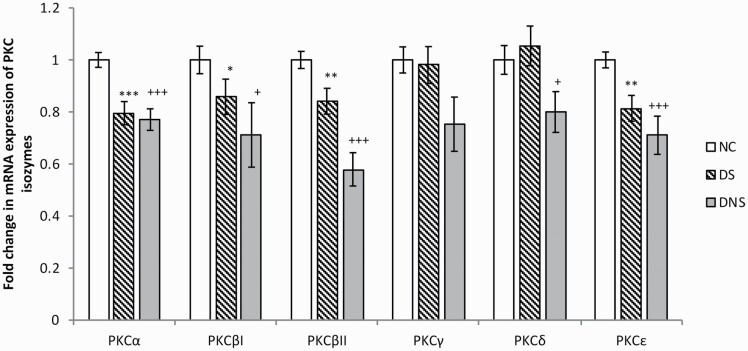
mRNA expression of protein kinase C (PKC) isozymes. The histogram represents fold change in mRNA expression of different PKC isozymes (PKC α, βI, βII, γ, δ, and ε) in prefrontal cortex (PFC) region of postmortem brains of control (NC), depressed suicide (DS), and depressed nonsuicide (DNS) subjects. Values are represented as mean fold change ± standard error of the mean (SEM). * (* =  ≤0.05, ** =  ≤0.01, *** = ≤0.001) and + ( + = ≤0.05, ++ = ≤0.01, +++ = ≤0.001) represent the statistically significant difference between NC-DS and NC-DNS, respectively.

Of the 6 PKC isozymes we studied, mRNA levels of 4 isozymes—PKCα (FC = 0.77, *P* = .0002), PKCβI (FC = 0.81, *P* = .047), PKCβII (FC = 0.82, *P* = .008), and PKCε (FC = 0.81, *P* = .004)—showed a significant decrease in the DS group compared with NC (FC = 1) (Figure 1). PKCγ and PKCδ isozymes showed no significant alteration in the brains of DS subjects. Further, PKCα, PKCβII, and PKCε also showed significant decreases after Bonferroni correction (*P* ≤ .008) in DS compared with NC subjects, but PKCβI failed the level of significance after correction.

We observed significant changes in PKCα (FC = 0.77, *P* = .0005), PKCβI (FC = 0.71, *P* = .047), PKCβII (FC = 0.58, *P* = .0001), PKCδ (FC = 0.80, *P* = .042), and PKCε (FC = 0.71, *P* = .0001) mRNA levels in DNS subjects compared with NC (FC = 1) (Figure 1). No changes were observed in PKCγ mRNA levels in DNS subjects. Further, the isozymes PKCα, PKCβII, and PKCε showed significant changes after Bonferroni correction (*P* ≤ .008), but PKCβI and PKCδ failed the level of significance after correction. Although we have observed reduced mRNA expression of all PKC isozymes in DNS subjects compared with DS subjects, these differences were not statistically significant.

### Protein Expression

**Figure 2.**
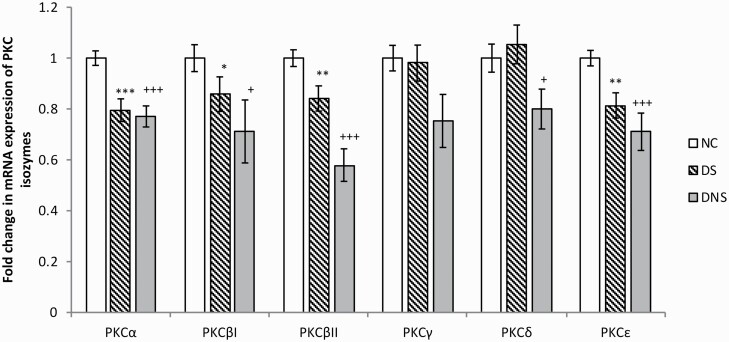
Cytosol and membrane protein expression of protein kinase C (PKC) isozymes. Representative blots of different PKC isozymes (PKC α, βI, βII, γ, δ, and ε) in (a) cytosol and (b) membrane fractions, and the histograms are presenting corresponding percentage change in protein expression of these PKC isozymes in (c) cytosol and (d) membrane fractions of prefrontal cortex (PFC) region of postmortem brains of normal control (NC), depressed suicide (DS), and depressed nonsuicide (DNS) subjects. Values are represented as mean percentage change ± standard error of the mean (SEM). *(* = ≤0.05, ** = ≤0.01, *** = ≤0.001) and + (+ = ≤0.05, ++ = ≤0.01, +++ = ≤0.001) represent the statistically significant difference between NC-DS and NC-DNS, respectively. The target protein expression was normalized by using β-actin as a house keeping gene.

To further study depression- and suicide-associated PKC isozyme abnormalities at the translational level, we performed western blotting and studied changes in protein expression of the 6 PKC isozymes in the cytosol and membrane fractions. The representative western immunoblots showing immunolabeling in the PFC of PKCα, PKCβI, PKCβII, PKCδ, PKCγ, and PKCε in the cytosol and membrane fractions of 2 NC, DNS, and DS subjects each are shown in Figure 2a–b, respectively. A trend toward decreased PKC isozymes expression in DS and DNS compared with NC can be observed from the immunoblots. Further analysis using MANCOVA with Bonferroni post-hoc test was performed to study the overall effect of diagnosis on the corrected model of protein expression in the 3 groups (i.e., NC, DS, and DNS). Cytosol protein expression of PKCβI (F[5,59] = 3.871, *P* = .005), PKCβII (F[5,59] = 3.474, *P* = .009), and PKCδ (F[5,59] = 3.391, *P* = .010) and membrane protein expression of PKCβI (F[5,58] = 2.367, *P* = .05) and PKCδ (F[5,58] = 2.342, *P* = .05) showed a significant decrease. Other isozymes—PKCα, PKCγ, and PKCε (both cytosol and membrane)—as well as membrane PKCβII protein expression showed no significant changes in overall effect between the 3 groups. The confounding variables such as age, PMI, and gender had no effects on protein expression in any of the PKC isozymes, whereas brain pH had a significant effect on PKCβI and PKCβII. We then compared the DS and DNS groups with respect to the NC group using pairwise comparisons and Bonferroni correction (*P* ≤ .008). Of 6 PKC isozymes, PKCβI (Cyto: PC = 0.79, *P* = .016; Memb: PC = 0.76, *P* = .015) and PKCβII (Cyto: PC = 0.75, *P* = .009; Memb: FC = 0.68, *P* = .015) showed significantly decreased protein expression in cytosol and membrane fraction from PFC of DS subjects (considering PC = 1 for NC). A significant decrease was also observed in the cytosol fraction of PKCδ protein (Cyto: PC = 0.75, *P* = .003; Memb: PC = 0.80, *P* = .30), but the change in membrane protein expression was not significant (Figure 2). No significant changes were observed in cytosol or membrane protein expression of PKCα, PKCγ, and PKCε isozymes.

Compared with NC subjects, cytosol and membrane protein expression of PKCβI (Cyto: PC = 0.78; *P* = .025; Memb: PC = 0.67, *P* = .002), cytosol protein expression of PKCβII (Cyto: PC = 0.75, *P* = .030; Memb: PC = 0.71, *P* = .089) and PKCδ (Cyto: PC = 0.61, *P* = .001; Memb: PC = 0.84, *P* = .633) was significantly downregulated in DNS subjects (Figure 2c–d). The change observed in other PKC proteins (i.e., membrane expression of PKCβII and PKCδ) was not significant compared with NC subjects. We observed no significant differences in the protein expression of PKC isozymes in DNS subjects compared with DS subjects.

### Correlation Between mRNA and Protein Expression

We determined the correlation between mRNA and protein expression of each PKC isozyme in all 3 groups. Although changes in mRNA and protein expression of each isozyme were in the same direction, there was no significant correlation between mRNA and protein expression of any PKC isozymes in DS, DNS, or NC subjects.

### Effect of Antidepressants

To examine the effect of antidepressants on the expression of PKC isozymes, we divided DS subjects into 2 groups:—those with antidepressants and those without antidepressants—and performed the *t* test. We observed no significant difference between these 2 groups, suggesting that antidepressants have no significant effect on the expression of PKC isoforms. Because 11 of 12 DNS subjects were on antidepressants, we did not analyze the effect of antidepressants in this group.

## Discussion

In this study, we examined the role of PKC signaling in the pathophysiology of depression and suicide by determining the gene and protein expression of PKC isozymes in PFC of postmortem brains of DS and DNS subjects. We found that the PKC isozyme’s mRNA (α, β, and ε) and protein (β and δ) expressions were significantly decreased in the PFC of DS subjects compared with NC subjects; however, there was no significant difference observed in the expression of PKCγ between these 2 groups at either mRNA or protein level. To examine if the changes we observed in DS subjects were related to either depression or suicide, we determined the expression of these PKC isozymes in another group of subjects who died due to natural causes and not by suicide (DNS). We found a significant decrease in mRNA expression of PKCα, PKCβI, PKCβII, PKCδ, and PKCε isozymes and a decrease in PKCβI, PKCβII, and PKCδ protein expression in DNS subjects. Changes in the mRNA and protein expression of PKC isozymes in both DS and DNS groups were very similar, with some minor differences. We found a decrease in the mRNA expression of PKCδ in the DNS but not in the DS group. In terms of protein expression, we found decreased PKCε in the cytosol and increased in the membrane fraction of the DNS but not the DS group. It is not clear if these minor differences between DS and DNS groups are related to depression or suicide. A possible explanation for the observed PKCε decrease in the cytosol and increase in the membrane fraction of the DNS group could be due to increased activation and phosphorylation of this PKC isozyme in the cytosol, leading to its translocation from the cytosol into the membrane. In summary, this study clearly shows the abnormal PKC signaling in depression and suicide.

We observed some dissociation in PKC expression between mRNA and protein levels. For example, PKCα and PKCε were significantly reduced at the mRNA level but not at the protein level in DS subjects compared with NC subjects. These dissociations can be explained based on other factors affecting RNA and protein synthesis, stability, and posttranslational modifications (Pandey et al., 2019).

PKC is a key regulatory enzyme present in various tissues, including the brain. PKC has been shown to be a family of at least 12 structurally related isozymes. All PKC isozymes possess 2 functional domains known as N-terminal regulatory domain and C-terminal catalytic domain. On the basis of molecular structure and enzyme characterization, the PKC family has been subgrouped into 3 classes known as conventional enzymes, which include PKCα, PKCβI, PKCβII, and PKCγ; novel PKC enzymes including PKCδ, PKCε, PKCθ, and PKCη; and atypical PKC isozymes including PKCζ and PKCλ/ί (Pandey et al., 1997; Abrial et al., 2011). Each enzyme is encoded by a unique gene. The conventional isozymes are phospholipid and calcium dependent, whereas the novel PKC isozymes do not require calcium for their activation. The atypical isozymes are unresponsive to phorbol esters but can be activated by phosphatidylserine. Most of the PKC isozymes are abundantly expressed in the central nervous system, especially in brain regions such as the PFC and hippocampus (Tanaka and Saito, 1992; Wetsel et al., 1992; Naik et al., 2000). In particular, the PKCε and PKCγ isoforms are shown to have higher expression in the brain compared with other tissues (Wetsel et al., 1992).

Because it has been shown that 5HT_2A_ receptors are abnormally high in postmortem brain of suicidal patients, it was important to examine if the biochemical effects of 5-HT_2A_ receptors, especially in signaling mechanisms, are also altered in the postmortem brains of suicide subjects. 5-HT_2A_ receptors are linked to the PI-PLC signaling cascade. In this signaling cascade, interaction of agonist such as serotonin with 5-HT_2A_ receptors or other G protein coupled receptors activates a Gq protein, which further activates PLC, which in turn cleaves the phosphatidylinositide-4,5 bisphosphate into DAG and IP_3_. IP_3_ in turn interacts with calcium channels on the endoplasmic reticulum to release stored calcium into cytoplasm. This increase in intracellular calcium then facilitates the translocation of PKC to the cell membrane, where it is activated by DAG (Gould and Manji, 2002; Steinberg, 2008; Cocco et al., 2015). On stimulation by DAG, PKC is recruited to the cell membrane and undergoes conformational changes, altering substrate binding and phosphorylation of its major substrates such as myristoylated alanine-rich protein kinase C substrate (MARCKS) and growth associated protein 43 (Steinberg, 2008; Callender and Newton, 2017). These substrates are involved in the pre- and postsynaptic regulation of neurotransmitter release and synaptic plasticity. PKC also activates cAMP response element binding protein, which causes transcription of many important genes such as brain-derived neurotrophic factor that plays an important role in the pathophysiology of depression (Shelton et al., 2009; Pandey, 2011).

Abnormal PKC signaling has been reported to be associated with different neuropsychiatric illnesses such as BP disorder, schizophrenia, depression, and suicide (Friedman et al., 1993; Pandey et al., 1998). The clinical studies reporting PKC signaling abnormalities in mental illnesses are performed in either peripheral tissues such as blood cells or in postmortem brains. Previous studies of PKC signaling in mood disorders mainly focused on PKC abnormalities in platelets and postmortem brains of BP patients. However, there is limited evidence of abnormalities of PKC and associated isozymes in depressed and suicide subjects, particularly in postmortem brains; this requires further exploration. In our own studies of PKC expression in platelets, we found a significant decrease in PKCα, PKCβI, and PKCβII in BP patients but not in platelets of MDD subjects (Pandey et al., 2002a).

The evidence for the involvement of PKC in depression has been provided by studies of PKC in the PFC of DS subjects by some investigators. Shelton et al. (2009) determined the protein expression of PKC isozymes in the PFC of MDD subjects, which included both suicidal and nonsuicidal subjects. They found lower levels of protein expression of PKCβI and PKCε in BA10 of melancholic MDD subjects compared with NC subjects. Further, they analyzed the data in terms of MDD-suicide, MDD-nonsuicide, and control subjects. However, there was no significant decrease in protein expression of either PKC isozymes in the MDD-suicide and MDD-nonsuicide subjects compared with NCs, which may be due to the small number of suicide and nonsuicide subjects (n = 12 and n = 8, respectively). On the other hand, they clearly observed a decrease in PKCβI and PKCε as mentioned before in the MDD subjects.

Hrdina et al. (1998) studied protein expression of various PKC isozymes in several brain areas such as BA9, BA10, amygdala, substantia nigra, and putamen of MDD suicide subjects (n = 10) and NC subjects. They observed a heterogeneous regional expression of PKC isozymes (α, β, γ, and ε), but no significant alteration was observed in MDD-suicide brains compared with control brain regions. Although Hrdina et al. (1998) did not observe significant differences in any of the PKC isozymes between depressed subjects and NC subjects, there was a nonsignificant trend towards decreased expression of PKCα, β, γ, and ε in BA9, PKCα, and ε in BA10, which may due to the small numbers of subjects (n = 10) they studied.

In 1 of our previous studies, we examined the protein and mRNA expression of PKC isozymes in the postmortem brain of teenage suicide and NC subjects. We examined PFC (BA9) and hippocampus of both sets of postmortem brains and observed a significant reduction in PKCα, PKCβ, and PKCγ proteins in both brain regions in teenage suicide subjects compared with controls (Pandey et al., 2004). The reduced protein expression was also associated with a decrease in respective isozyme mRNA expression and reduced catalytic activity of PKC (Pandey et al., 2004). In another study, we examined the PKC activity and binding in teenage suicide victims and found a significant reduction in PKC binding/recognition sites in both membrane and cytosol fractions of cortex compared with NC subjects (Pandey et al., 1997).

Strong evidence for the PKC role in depression has also been presented by some animal studies. The role of the PKC in depression is also derived from studies of animal models of depression; for example, repeated administration of antidepressants significantly attenuated PKC activity and PKCβI expression in stressed rats (Mann et al., 1995). Also, administration of PKC activators, such as phorbol 12-myristate 13-acetate to rats resulted in an antidepressant-like activity as indicated by a reduction of immobility (Abrial et al., 2011). Some studies have determined the effect of chronic and acute treatment with antidepressants on PKC and PKC pathways. For example, it was observed that antidepressant treatment increased PKC activity and MARCKS phosphorylation in the rat brain (Szabo et al., 2009). However, chronic antidepressant treatment has also been shown to decrease PKC activity and cause inhibition of PKCγ and PKCδ isoforms in the rat brain (Mann et al., 1995; Rausch et al., 2002). The evidence that PKC may also be involved in depression is based on the observation that treatment with antidepressants increased phosphorylation of PKC substrates such as GAP-43 and MARCKS (Szabo et al., 2009; Kim et al., 2010). Further, a recent study demonstrated reduced PKC levels in depressed rats, which were reversed by treatment with antidepressant-like agent SiNiSan and standard antidepressant fluoxetine (Shen et al., 2020). Further, a specific inhibition study of PLC-PKC cascade in mice suggested that depressive behaviors can be modulated by inhibiting some PKC isozymes. Inhibition of PKCγ by chelerytrine and calphostin C or knockdown using antisense RNA strategy exhibited antidepressant-like effects (Galeotti and Ghelardini, 2011). Abrial et al. (2013) found that chronic administration of the PKC inhibitor tamoxifen reduced the hyperlocomotion administration of chelerytrine caused depressive-like behavior in forced swim test resulting in reduced cell proliferation in the dentate gyrus of the hippocampus. Both the clinical (platelet and postmortem) and animal (antidepressant treatment as well as animal models) studies thus indicate that abnormalities of PKC are not only associated with BP disorders but may also be associated with depressive illness.

The biological effects of PKC such as synaptic plasticity may be related to the phosphorylation of several downstream targets of the PKC such as neurogranin, neurotrophic factors, GAP-43, and MARCKS. MARCKS and GAP-43 have been shown to play a role in depression and have been studied in postmortem brains of depressed and suicidal patients (Hrdina et al., 1998; Pandey et al., 2003). A decrease in PKC may reduce phosphorylation of MARCKS, which then further alters its translocation. We have observed a significant decrease in PKC-mediated MARCKS phosphorylation in DS subjects along with high membrane MARCKS protein expression, suggesting altered translocation from membrane to cytoplasm (Pandey et al., 2003). Alterations in MARCKS expression in mood disorders are also suggested by other studies such as lithium-induced decrease in MARCKS expression in rat hippocampus (Lenox et al., 1992; Wang et al., 2000) and increased MARCKS expression in platelets of BP patients (Pandey et al., 2002a). GAP-43, the other downstream target of PKC, was studied by (Hrdina et al., 1998), who observed a significant decrease in its mRNA as well as protein expression in PFC of postmortem brains of suicide subjects compared with NCs. However, they did not observe significant changes in any of the PKC isozymes. Additionally, calcium-mediated phosphorylation of GAP-43 by PKC is crucial in stabilizing new synapses and synaptic plasticity (Holahan, 2017).

These clinical and preclinical studies thus suggest that PKC alteration has been strongly associated with mood disorders, suicide, and depression. Although many studies have reported downregulation of PKC levels or activity with few reports of enzyme-specific alterations, the mechanism for decreased PKC isozyme levels is still not clear. It could be speculated to be due to the following reasons: (1) agonists such as DAG or phorbol esters induce activation and cytosol to membrane translocation as well as proteolytic cleavage of PKC, which could result in reduction of PKC protein levels; (2) it could also be the result of compensatory mechanism against prolonged activation of G-coupled receptors due to stress, depression, or suicide. Continuous activation of receptors leads to prolonged stimulation of PKC, which was found to promote translocation and subsequent degradation of PKC isozymes; (3) hypothalamic-pituitary-adrenal axis abnormalities associated with depression and suicide induce downregulation of PKC and some of its isozymes and thus may contribute to the observed decline in PKC isozymes; and (4) another speculated factor could be the effect of antidepressants, specifically the DNS group subjects were on chronic antidepressant treatment. Lithium, valproate, and other antidepressants have been shown to modulate PI-PKC signaling by reducing PKC levels.

The current study is the most comprehensive study examining the role of depression and suicidality on PKC isozymes in postmortem brain of adult DS and nonsuicide subjects. Abnormalities in PKC signaling may affect the downstream target molecules and transcription factors; for example, one of the important targets of PKC is cAMP response element binding, which binds with different CREs and regulates the expression of various genes, including the ones that are implicated in pathophysiology of depression (Shelton et al., 2009). Thus, sufficient evidence of particular PKC isozyme abnormalities, as provided by the results of the current study, may help in the development of neurotherapeutic drugs or other treatment approaches specifically targeting the PKC signaling system and could help to improve the effectiveness of treatments for depression treatment, thus reducing suicide attempts.

## Supplementary Material

Supplementary data are available at *International Journal of Neuropsychopharmacology (IJNPPY)* online.

Supplementary Table 1. Detailed characteristics of subjects in the NC, DS, and DNS groups.

pyab003_suppl_Supplementary_Table_1
