# Supplementary material for: Dysregulation of Protein Kinase C in Adult Depression and Suicide: Evidence From Postmortem Brain Studies
Source: Int J Neuropsychopharmacol. 2021 Jan 30;24(5):400–8. doi: 10.1093/ijnp/pyab003 (PMC8130206; doi:10.1093/ijnp/pyab003)
Supplement: pyab003_suppl_Supplementary_Table_1 [file pyab003_suppl_supplementary_table_1.doc]

**Supplementary Table 1.** **Demographic Characteristics of Subjects**

|  | **Group** | **Age (years)** | **Race** | **Gender** | **PMI (hours)** | **Brain**  **pH** | **Cause of Death** | **Psychotropic Drugs**  **(at the time of death)** | **Psychiatric Diagnosis** |
| --- | --- | --- | --- | --- | --- | --- | --- | --- | --- |
|  |  |  |  |  |  |  |  |  |  |
| **Normal Control Subjects ^a^** | | | | | | | | | |
| 1. | CONTROL | 19 | Black | Male | 11 | 6.9 | GSW | None | Normal |
| 2. | CONTROL | 22 | Black | Male | 19 | 6.9 | GSW | None | Normal |
| 3. | CONTROL | 42 | White | Female | 23 | 7.2 | Pneumonia | None | Normal |
| 4. | CONTROL | 37 | Black | Male | 5 | 7.1 | ASCVD | None | Normal |
| 5. | CONTROL | 31 | Black | Male | 8 | 7.2 | GSW | None | Normal |
| 6. | CONTROL | 46 | Black | Male | 9 | 7.1 | Multiple injuries | None | Normal |
| 7. | CONTROL | 33 | White | Male | 15 | 7.0 | GSW | None | Normal |
| 8. | CONTROL | 48 | White | Male | 26 | 6.9 | ASCVD | None | Normal |
| 9. | CONTROL | 40 | White | Female | 7 | 7.0 | ASCVD | None | Normal |
| 10. | CONTROL | 23 | Black | Male | 15 | 6.8 | GSW | None | Normal |
| 11. | CONTROL | 83 | White | Male | 20 | 7.1 | ASCVD | None | Normal |
| 12. | CONTROL | 65 | Black | Female | 23 | 6.9 | ASCVD | None | Normal |
| 13. | CONTROL | 35 | White | Male | 24 | 6.9 | Crush injury to abdomen and chest | None | Normal |
| 14. | CONTROL | 52 | White | Male | 30 | 7.3 | ASCVD | None | Normal |
| 15. | CONTROL | 37 | White | Male | 24 | 7.0 | ASCVD | None | Normal |
| 16. | CONTROL | 45 | White | Male | 22 | 7.3 | ASCVD | None | Normal |
| 17. | CONTROL | 26 | White | Male | 12 | 6.9 | Arrhythmia | None | Normal |
| 18. | CONTROL | 47 | White | Male | 10 | 7.0 | ASCVD | None | Normal |
| 19. | CONTROL | 31 | White | Male | 16 | 7.2 | MVA | None | Normal |
| 20. | CONTROL | 60 | White | Male | 15 | 7.1 | Accidental drowning | None | Normal |
| 21. | CONTROL | 28 | White | Male | 13 | 6.8 | Electrocution | None | Normal |
| 22. | CONTROL | 45 | White | Female | 16 | 6.9 | Cardiac arrhythmia | None | Normal |
| 23. | CONTROL | 62 | White | Male | 19 | 7.0 | Cardiac arrest | None | Normal |
| 24. | CONTROL | 53 | White | Male | 15 | 6.9 | ASCVD | None | Normal |
|  |  |  |  |  |  |  |  |  |  |
|  |  |  |  |  |  |  |  |  |  |
| **Depressed Suicide Subjects ^b^** | | | | | | | | | |
| 1. | SUICIDE | 27 | White | Male | 24 | 7.0 | GSW | None | MDD, Ethanol abuse |
| 2. | SUICIDE | 44 | White | Female | 11 | 7.2 | Drug overdose | Nortriptyline | MDD, Ethanol abuse |
| 3. | SUICIDE | 36 | White | Female | 10 | 7.1 | GSW | None | MDD |
| 4. | SUICIDE | 24 | White | Male | 7 | 7.1 | GSW | Ethanol | MDD |
| 5. | SUICIDE | 43 | White | Male | 12 | 7.0 | Drug Overdose | None | MDD, Polysubstance Abuse |
| 6. | SUICIDE | 53 | White | Male | 23 | 6.9 | Jumped from height | None | MDD |
| 7. | SUICIDE | 41 | White | Female | 27 | 7.1 | Drug Overdose | Amitriptyline, Desipramine, Nortriptyline, Ethanol | MDD, Ethanol abuse |
| 8. | SUICIDE | 22 | Black | Female | 16 | 7.3 | Drug overdose | None | MDD |
| 9. | SUICIDE | 46 | White | Female | 21 | 6.9 | Drug overdose | Amitriptyline, Desipramine, Ethanol | MDD |
| 10. | SUICIDE | 36 | White | Female | 18 | 7.2 | GSW | None | MDD |
| 11. | SUICIDE | 38 | White | Male | 24 | 7.0 | Drug overdose & Ethanol overdose | Ethanol | MDD, Ethanol abuse |
| 12. | SUICIDE | 46 | White | Female | 16 | 6.8 | Drug overdose /  Nortryptyline Intoxication | Nortriptyline | MDD, Panic disorder with agoraphobia |
| 13. | SUICIDE | 23 | White | Male | 12 | 7.0 | Hanging | Paroxetine | MDD |
| 14. | SUICIDE | 18 | White | Male | 17 | 6.3 | Hanging | None | MDD |
| 15. | SUICIDE | 30 | White | Male | 17 | 7.1 | Hanging | Venlafaxine | MDD |
| 16. | SUICIDE | 19 | White | Male | 18 | 6.2 | CO intoxication | Ethanol, CO | MDD, Ethanol abuse, Polysubstance abuse |
| 17. | SUICIDE | 44 | White | Female | 30 | 7.2 | Drug overdose, Ethanol intoxication | Fluoxetine, Ethanol | MDD, Ethanol abuse, Opioid abuse |
| 18. | SUICIDE | 74 | White | Female | 27 | 7.0 | Venlafaxine overdose | Venlafaxine, Ethanol | MDD, Ethanol abuse |
| 19. | SUICIDE | 25 | White | Male | 14 | 6.8 | Hanging | Ethanol | MDD |
| 20. | SUICIDE | 23 | Black | Male | 23 | 6.9 | Hanging | None | MDD |
| 21. | SUICIDE | 63 | White | Male | 19 | 6.9 | Drug overdose, Ethanol intoxication | Ethanol | MDD |
| 22. | SUICIDE | 67 | White | Male | 22 | 7.0 | GSW | Fluoxetine, Venlafaxine | MDD |
| 23. | SUICIDE | 40 | White | Female | 20 | 7.0 | Drug overdose | Alprazolam | MDD |
| 24. | SUICIDE | 53 | White | Male | 26 | 7.1 | Suicide by stab wound | Sertraline | MDD |
|  |  |  |  |  |  |  |  |  |  |
|  |  |  |  |  |  |  |  |  |  |
| **Depressed Non-Suicide Subjects ^c^** | | | | | | | | | |
| 1. | Non-suicide depressed | 65 | White | Male | 14 | 6.9 | ASCVD | None | MDD |
| 2. | Non-suicide depressed | 55 | Black | Female | 8 | 6.4 | ASCVD | Fluoxetine, Ethanol | MDD, Polysubstance abuse |
| 3. | Non-suicide depressed | 71 | White | Male | 4 | 6.3 | ASCVD | Bupropion | MDD |
| 4. | Non-suicide depressed | 74 | Black | Female | 7 | 6.7 | ASCVD | Paroxetine, Thioridazine | MDD |
| 5. | Non-suicide depressed | 14 | White | Male | 11 | 7.0 | MVA | Sertraline | MDD, Polysubstance abuse |
| 6. | Non-suicide depressed | 39 | White | Male | 36 | 6.8 | Fatty Liver | Thioridazine | MDD |
| 7. | Non-suicide depressed | 46 | Black | Male | 20 | 7.1 | Seizure d/o | Fluoxetine, Risperidone | MDD |
| 8. | Non-suicide depressed | 59 | White | Male | 20 | 7.0 | ASCVD | Sertraline | MDD, Ethanol dependence |
| 9. | Non-suicide depressed | 46 | White | Female | 23 | 6.9 | Mixed Drug intoxication | Bupropion, Lamotrigine | MDD, Ethanol abuse, Polysubstance abuse |
| 10. | Non-suicide depressed | 29 | White | Female | 22 | 6.9 | Obesity,  Cardiomegaly | Fluoxetine, Norfluoxetine | MDD |
| 11. | Non-suicide depressed | 49 | White | Male | 24 | 7.1 | ASCVD | Desmethylsertraline | MDD |
| 12. | Non-suicide depressed | 47 | White | Female | 26 | 6.5 | Diabetic ketoacidosis | Fluoxetine | MDD |

ASCVD, atherosclerotic cardiovascular disease; CO, carbon monoxide; GSW, gunshot wound; MDD, major depressive disorder; MVA, motor vehicle accident

^a^. Mean ± SD age is 42.08 ± 15.35 years; PMI is 16.54 ± 6.56 hours; brain pH is 7.02 ± 0.15; 7 Black, 17 White; 20 Males, 4 Females

^b^. Mean ± SD age is 38.96 ± 15.40 years; PMI is 18.92 ± 6.02 hours; brain pH is 6.96 ± 0.25; 2 Black, 22 White; 14 Males, 10 Females

^c^. Mean ± SD age is 49.50 ± 17.18 years; PMI is 17.92 ± 9.32 hours; brain pH is 6.80 ± 0.27*; 3 Black, 9 White; 7 Males, 5 Females

*p< 0.05 (compared with NC group)
